# HIV and SARS-CoV-2 Pathogenesis and Vaccine Development

**DOI:** 10.3390/v14122598

**Published:** 2022-11-22

**Authors:** Herve Fleury

**Affiliations:** Université de Bordeaux et CNRS, 33076 Bordeaux, France; h.fleury@icloud.com

Although both HIV and SARS-CoV-2 are associated with pandemics, they are transmitted differently. 

HIV is transmitted mainly through sexual contact and is associated with a slowly evolving disease from primary infection to AIDS. It has progressively affected all continents and continues to present a major public health problem. Many research programs have been devoted to HIV research with one principal victory: antiretroviral therapy, from AZT to protease and integrase inhibitors. Despite this, in the almost 40 years after its discovery, there is still no preventive vaccine against HIV. Furthermore, a viral cure has not been found for chronically infected patients through different approaches, including therapeutic vaccines. As such, research on HIV still remains a priority for the future.

SARS-CoV-2 is transmitted through a respiratory route. Emerging at the end of 2019 in China, the virus quickly evolved into a global pandemic, much like the H1N1_pdm_ flu virus. Like SARS-CoV and MERS-CoV, it is a coronavirus, the reservoir of which is the bat. A tsunami of research has been dedicated to this virus, and significant progress has been made, particularly via vaccines and antiretroviral drugs. However, there are still questions to address regarding pathophysiology and vaccine efficacy in relation to viral replication.

This Special Issue was launched in 2020; 19 articles have been accepted for publication; they range from original articles to reviews.

Regarding HIV, several topics are addressed: the funding of biomedical research for HIV vaccine in USA ([1] Shapiro et al), basic research on immunology on an SIV model ([2] Trovato et al.), modulation of HIV replication in macrophages ([3] Biswas et al.), multi-envelope HIV-1 vaccine design ([4] Sealy et al.), mRNA vaccine and their potential efficiency for an HIV cure ([5] Esteban et al.), and Elispot response of ART-treated patients to a potential HIV vaccine ([6] Fleury et al.)

Regarding SARS-CoV-2, we identify basic research reports on spike/ACE2 interaction ([7] Lapaillerie et al.), plant-produced viral proteins for vaccine preparation ([8] Mamedov et al.), molecular insights of SARS-CoV-2 and consequences on efficacy of current vaccines ([9] Rotondo et al.), peritoneal administration of a subunit vaccine ([10] Jearanaiwitayakul et al.), fatal neurodissemination of SARS-CoV-2 in an animal model ([11] Carossino et al.), natural and experimental SARS-CoV-2 infection in domestic and wild animals ([12] Meekins et al.), and virus eradication and synthetic biology ([13] Tournier et al.)

There are clinical reports, some of them bridging the two topics: obstetric outcome in SARS-CoV-2 infected pregnant women ([14] Cruz-Lemini et al.), COVID 19 vaccines for HIV infected patients ([15] Plummer et al.), severe COVID-19 infection in HIV-infected patients on ART ([16] Mazzitelli et al.), mRNA and recombinant adenovirus vaccines against SARS-CoV-2 in HIV-infected patients ([17] Garbuglia et al.), multiple infection with SARS-CoV-2, HIV and Mycobacterium tuberculosis ([18] González-Domenech et al.), Cytomegalovirus and response to vaccines in HIV-infected patients ([19] Royston et al.).

All articles are of great interest to the readers of *Viruses*, and I would like to thank the authors and research groups for their contributions to this Special Issue. 

My thanks also to the editors and reviewers who help maintain and reinforce the standards of this journal. Best regards to all of you.
